# Dysphagia Evaluation after Stroke through Intervention with oral neuromuscular training for REhabilitation of swallowing dysfunction (DESIRE): study protocol for a multicenter randomized controlled trial

**DOI:** 10.1186/s13063-026-09778-1

**Published:** 2026-05-19

**Authors:** Patricia Hägglund, Liza Bergström, Johanna Forsgren, Thorbjörn Holmlund, Per Liv, Eva Levring Jäghagen, Vesna Mirkoska, Mihaela Romanitan, Jenny Selg, Per Wester

**Affiliations:** 1 Speech and Language Pathology, Department of Clinical Sciences, Faculty of Medicine, Umeå University, Umeå, Sweden; 2 Speech and Language Pathology, Division of Neurology, Department of Clinical Sciences, Karolinska Institute, Danderyd Hospital, Stockholm, Sweden; 3 Otorhinolaryngology, Department of Clinical Sciences, Faculty of Medicine, Umeå University, Umeå, Sweden; 4 Department of Public Health and Clinical Medicine, Umeå University, Umeå, Sweden; 5 Oral and Maxillofacial Radiology, Department of Odontology, Faculty of Medicine, Umeå University, Umeå, Sweden; 6 Department of Clinical Science and Education, Karolinska Institute, Södersjukhuset, Internal Medicine, Stockholm, Sweden; 7 Department of Clinical Sciences, Karolinska Institutet, Danderyd Hospital, Stockholm, Sweden

**Keywords:** Stroke, Dysphagia, Oral neuromuscular training, Rehabilitation, Sensor-equipped oral device, Randomized controlled trial, Standard care, Clinical outcomes, Quality of life

## Abstract

**Background:**

Stroke affects over 1 million people in Europe and 25,000 in Sweden annually, with high mortality and disability rates. Dysphagia, a common post-stroke issue, occurs in over 50% of patients in the acute phase. Evidence-based studies are lacking on rehabilitative interventions to improve post-stroke dysphagia. Current management focuses on compensatory strategies, but oral neuromuscular training shows promising results. Challenges to rehabilitation programs include poor adherence and the need for effective monitoring technologies. The primary objective of the current trial is to investigate the impact of oral neuromuscular training with a sensor-equipped oral device on post-stroke dysphagia.

**Methods:**

This national, multicenter, investigator-initiated, randomized, parallel-group trial compares the effect of oral neuromuscular training versus standard care in patients with post-stroke dysphagia. We will enroll 336 consecutive participants who have no prior history of dysphagia and exhibit persistent dysphagia 3 months following the onset of ischemic stroke or intracerebral hemorrhage. Participants will be randomly assigned in a 1:1 ratio to receive either a 12-week intervention consisting of standard routine care combined with oral neuromuscular training (using an oral device with sensors for adherence feedback) or current routine care alone. Standard routine care includes compensatory strategies such as bolus modification, adapted swallowing postures, and maneuvers. The primary outcome is the degree of dysphagia at end of intervention, assessed with Flexible Endoscopy Evaluation of Swallowing (FEES) by blinded evaluators, and analyzed with ordinal regression models. Secondary outcomes are changes in nutritional status, pneumonia, mortality, and quality of life after a 12-week intervention and at 6-month follow-up.

**Discussion:**

This first randomized clinical trial in stroke rehabilitation on neuromuscular oropharyngeal training with sensors to facilitate adherence and comprehensive outcome measures will enable evaluation of this treatment with the potential for significant clinical impact for patients with dysphagia after stroke.

**Trial registration:**

ClinicalTrials.gov, identifier: NCT02960737. First registered on 11 November 2016 and later modified to the present multicenter study design on 22 August 2023.

## Introduction

### Background and rationale {9a}

Stroke is a major public health issue, affecting over 1 million people in Europe and 25,000 in Sweden each year, with deaths in one-fifth and persistent disability in almost half of the survivors [1]. It is the second leading cause of death worldwide and the foremost contributor to dysfunction and disability among adults. Common post-stroke complications include hemiparesis, sensory loss, aphasia, dysphagia, hemianopia, and neuropsychological symptoms. In Europe, stroke care costs are estimated to rise from €60 to €86 billion by 2040, with Oxford University suggesting actual costs may be higher due to excluded social and home care services [2]. In Sweden alone, the societal cost of strokes surpasses €1.8 billion each year, placing significant pressure on the healthcare system.

*Post-stroke dysphagia*: Dysphagia refers to difficulties in swallowing and transporting food, drink, saliva, and medications from the mouth to the stomach. Stroke is considered the leading cause of dysphagia among adults, with an estimated incidence of over 50% in the acute phase [3]. Dysphagia is further reported in 17% of patients at 28 days [4] and in 10–15% at 6 months after a stroke [5]. Dysphagia is related to various secondary complications, including malnutrition, dehydration, and aspiration pneumonia, resulting in impaired life quality and increased risk of early mortality [3, 6, 7].

During the acute phase of a stroke, guidelines recommend that all patients undergo a swallowing assessment using a validated screening tool prior to any oral intake [8–11]. If dysphagia is suspected, a thorough evaluation should be performed using Flexible Endoscopic Evaluation of Swallowing (FEES) [10]. This approach is essential for ensuring accurate diagnostics, as instrumental assessments are acknowledged for their superior sensitivity in detecting dysphagia.

Current approaches to managing post-stroke dysphagia primarily focus on compensatory strategies, such as adjusting head and neck posture and modifying the consistency of food and fluid intake [12]. While these methods can help mitigate the risk of aspiration, they do not address the underlying swallowing impairment. In severe cases, patients may require artificial nutrition through feeding tubes, which is associated with increased care demands, hospitalization, and costs.

Various attempts with direct intervention on swallowing physiology and post-stroke dysphagia with rehabilitative strategies such as pharyngeal electrical stimulation, tactile stimulation, transcranial direct current stimulation, and transcranial magnetic stimulation have all been without significant positive effects [12, 13]. Oral neuromuscular training with an oral device is a promising alternative. A small randomized controlled trial showed improvement in post-stroke dysphagia [14], and positive results have been observed among older people with dysphagia [15].

This intervention aims to directly improve swallowing physiology and function, potentially offering a more effective solution than compensatory strategies alone. However, larger, more rigorous clinical trials are needed to establish the efficacy of this novel approach. This area is highlighted for research and development in the Swedish National Stroke Guidelines, underscoring its importance.

One of the major challenges in managing post-stroke dysphagia is the low adherence to rehabilitation programs. Studies have reported that less than 50% of patients maintain engagement with home-based stroke therapy within the first month after hospital discharge [16]. Improving adherence is crucial, as it is directly linked to better patient outcomes and reduced healthcare costs. To address this issue, researchers have explored the use of sensor technologies. These technologies provide real-time monitoring of patient adherence, potentially enabling more accurate tracking and support for patients while minimizing the risks associated with biased or inaccurate self-reporting [17].

Post-stroke dysphagia presents significant challenges requiring effective, evidence-based interventions to enhance swallowing function and mitigate associated risks. Continued research into novel treatment strategies and adherence monitoring is crucial to address these unmet needs and improve outcomes for stroke survivors.

### Explanation for the choice of comparator {9b}

Compensatory strategies are considered standard care for post-stroke dysphagia [12].

### Objectives {10}

The primary objective of this study is to investigate the effect of a 12-week intervention with an oral neuromuscular training device in stroke patients with persistent dysphagia in the sub-acute phase. Secondary objectives are to investigate the treatment effect on aspiration pneumonia, swallowing-related quality of life, and death.

## Methods: patient and public involvement, and trial design

### Patient and public involvement {11}

Patient and public involvement has played a central role in the development of this trial. Years of clinical experience and ongoing dialogue with individuals with post-stroke dysphagia as well as their relatives have highlighted the substantial impact of dysphagia on daily life, social participation, and overall well-being, underscoring the importance of user involvement to optimize treatment adherence and outcomes. To improve the study design, feedback was collected from patients and relatives, and contact was established with regional stroke associations at participating sites. Patient representatives have been recruited to the Steering Committee ensuring continuous patient input throughout the planning, conduct, and evaluation phases of the trial. Their contributions have directly shaped key aspects of the study, including refinement of the training method, incorporation of a sensor to support compliance, involvement of relatives in the treatment process, and extension of the training period to 3 months based on user-reported needs and clinical experience. In response to patient preferences for continued follow-up, a 6-month post-treatment assessment has also been incorporated. Patient representatives will remain actively involved throughout the trial to ensure that the study remains aligned with patient priorities and experiences.

### Trial design {12}

The DESIRE is designed as a multicenter, national, investigator-initiated, randomized, parallel-group, superiority trial to compare the effect of 12-week oral neuromuscular training versus standard care in patients with persistent post-stroke dysphagia and with the primary endpoint of degree of swallowing dysfunction immediately after the intervention period.

## Methods: participants, interventions, and outcomes

### Trial setting {13}

Participants will be recruited from multiple sites across Sweden, including academic and regional hospitals and outpatient clinics, to achieve the target sample size. To date, recruitment of participants is ongoing at nine sites. Further sites will be recruited. Study assessments will take place at each site or at a healthcare center close to the participant’s home, whereas the intervention will be performed daily by the participant in their home or care unit/nursing home.

### Characteristics of the people who are needed for the trial

The expected characteristic of the people that will be included in the study is displayed in Table 1.

**Table 1:** Expected demographic characteristics of the participants

Characteristic	The people we would expect to see included
Age	Individuals aged 18 years and above; with mean age 75 based on age distribution in the Swedish National Stroke Registry [1]
Sex	Female and male
Gender	Both women and men; with a slightly higher proportion of men, consistent with sex distribution in the Swedish National Stroke Registry [1]
Race, ethnicity, and ancestry	Predominantly individuals of European descent, reflecting the Swedish population; minority representation expected to be low based on national demographics
Socioeconomic status	Participants from varied socioeconomic backgrounds; distribution expected to reflect national patterns, with most in middle-income categories
Geographic location	Nationwide recruitment including rural, suburban, and urban areas across southern, central, and northern Sweden
Other characteristics relevant to the trial	Not applicable

### Eligibility criteria for participants {14a}

**Fig. 1:**
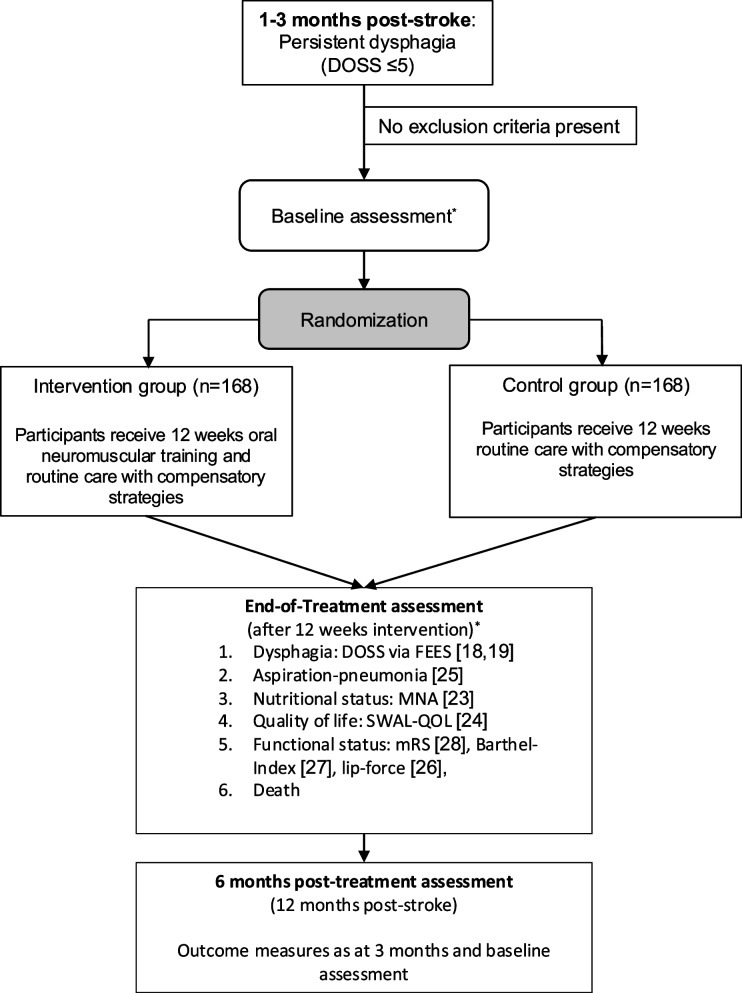
Study inclusion and procedure of the DESIRE study. *The same assessments are performed at baseline as at 12 weeks (post-intervention) and 6 months post-intervention. Abbreviations: DOSS = Dysphagia Outcome Severity Scale; FEES = Flexible Endoscopic Evaluation of Swallowing; MNA = Mini Nutritional Assessment; SWAL-QOL = Swallowing Quality of Life Questionnaire; mRS = modified Rankin Scale

Eligibility criteria are consecutive patients with stroke (ischemic or hemorrhagic) with persistent dysphagia (defined as Dysphagia Outcome Severity Scale, DOSS, ≤5 on FEES assessment) [18, 19] at 1–3 months (4–12 weeks) post-stroke onset. Exclusion criteria: previously known dysphagia; unable or unwilling to give informed consent or to cooperate. The study inclusion and procedure are depicted in Fig. 1.

### Eligibility criteria for sites and those delivering interventions {14b}

Eligibility criterion for individuals who will perform the assessments is that they are trained in the consent process, study protocol, and are FEES competent (i.e., speech-language pathologist and/or otolaryngologist).

### Who will take informed consent? {32a}

Trained investigators at each site will introduce the trial to patients at the standard FEES assessment at 1–3 months post-stroke, providing them with written information about the study. Subsequently, the investigators will facilitate discussions about the trial, giving patients the opportunity to ask questions and seek clarification on the provided materials. The investigators will be certified in Good Clinical Practice (GCP) [20] and the Declaration of Helsinki [21].

For patients with aphasia or those unable to write after a stroke, verbal consent will be sought for participation, as witnessed by an independent observer such as a staff member, relative, or friend, who will then sign and date the form.

### Additional consent provisions for collection and use of participant data and biological specimens {32b}

N/A as we will not collect biological specimens in this trial and no additional study is planned. Any future use of participant data beyond the scope of this protocol would require new ethical approval and additional consent.

## Intervention and comparator

### Intervention and comparator description {15a}

**Fig. 2:**
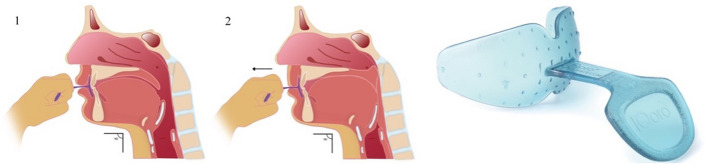
Oral neuromuscular training (left). The device is placed pre-dentally behind closed lips. The device is then pulled straight forward for 5–10 s, keeping the device in place with the lips by activating the orofacial, oral, and pharyngeal muscles. The training is repeated three times, with a 3-s rest in-between. Oral device (right)

The intervention group will be given intensive oral neuromuscular training of orofacial and pharyngeal muscles with an oral device (IQoro® Myoroface, Sweden). Oral neuromuscular training involves enhancing sensory input and strengthening the facial, oral, and pharyngeal muscles. An oral device is positioned pre-dentally behind closed lips and is then pulled straight forward for a duration of 5–10 s, keeping the device in place with the lips by activating the orofacial, oral, and pharyngeal muscles (see Fig. 2). This exercise is repeated three times, with a rest period of 3 s between each repetition. The training is conducted three times daily, for 12 weeks. Additionally, the intervention group will receive individualized compensatory strategies equivalent to those provided to the control group.

The control group will be given individualized instruction on compensatory strategies to compensate for their specific swallowing dysfunction. For example, posture considerations for head, neck, and body during swallowing, swallowing maneuvers, and modification of bolus volume, consistency, and texture.

### Criteria for discontinuing or modifying allocated intervention/comparator {15b}

There are no provisions for modifying the allocation of trial arms. Participants have the right to withdraw from the study at any time without providing an explanation, and their future care will remain unaffected. All data collected prior to withdrawal will be retained and included in the analyses.

### Strategies to improve adherence to intervention/comparator {15c}

To improve participant adherence, a sensor is attached to the oral device to register the treatment compliance/adherence, i.e., the frequency (newton) and duration (seconds) of training—which will give feedback to the investigator, the patient, and for data collection. Frequency and duration of the training is registered while the participant is using the oral device and is uploaded via an app using Bluetooth for feedback on the training session.

### Concomitant care permitted or prohibited during the trial {15d}

Throughout the trial, participants in both groups will not be allowed to employ any other active training methods, except for modifying food consistencies to enhance swallowing.

### Ancillary and post-trial care {34}

No formal post care provision is planned in the study. Participants with persistent dysphagia at follow-up 6-month post-intervention (i.e., 12 months post-stroke) will be treated according to the clinical routine at each site.

### Outcomes {16}

#### Primary outcome

- Post-intervention degree of swallowing dysfunction, measured immediately after the completion of the 12-week oral neuromuscular training intervention or use of compensatory strategies (end-of-treatment assessment); examined transnasally with a FEES, assessed using the DOSS [18, 19]. DOSS is an internationally widely used scale for measuring swallowing safety and efficacy on an ordinal scale and is based on the International Classification of Functioning, Disability and Health (ICF) classification. DOSS lists objective criteria on a 7-point Likert scale to systematically rate the functional swallowing severity from 1 (severe dysphagia and total dependency of artificial nutrition) to 7 (normal swallowing function and normal diet) based on symptoms observed during a FEES and impact on eating/drinking function. Subsequently, the DOSS results are directly transferable into recommendations for the patients regarding the diet level (tube feeding, modified diet, or no restriction), independence level (grade of assistance during mealtime), and type of nutrition the patient can swallow safely (liquid, thickened bolus, etc.). The scale has shown high validity and excellent inter- and intra-rater reliability [18, 19]. To account for participants who are too fragile to undergo the FEES examination, a composite variable strategy [22] will be employed to mitigate bias. Specifically, if a DOSS score is missing because a participant is judged to have severe swallowing dysfunction or frailty preventing assessment, a DOSS value of 0 (representing the worst outcome) will be imputed. This procedure is described in greater detail in the section: “Methods in analysis to handle protocol non-adherence and any statistical methods to handle missing data.”

#### Secondary outcomes

The intervention and the control group will be compared at end-of-treatment and at 6-month follow-up, adjusted for baseline values on the following secondary outcomes:

- Nutritional status [23] is measured with the Mini Nutritional Assessment Short Form (MNA-SF). The MNA-SF contains 6 items with a total score ranging from 0 to 14; a score of ≥12 points indicate that a person is well-nourished, 8–11 points indicate a risk of malnutrition, and <7 points indicate that the person is malnourished. MNA has been shown to have high sensitivity and specificity (96% and 98%, respectively) and significant agreement between observers (*K* = 0.51) and the MNA-SF has been shown to be a stand-alone nutritional assessment. The total score will be analyzed as an ordinal variable without categorization and compared between group.
- Swallowing-related quality of life is measured with the Swallowing Quality of Life Questionnaire (SWAL-QOL) [24], which is a patient-reported outcome measure (PROM) and consists of 44 items on a 5-point Likert scale that assesses 10 quality-of-life concepts: burden, eating duration, eating desire, food selection, communication, fear, and mental health. It further contains a dysphagia clinical symptom scale (symptom score). Scores for each domain are calculated using a linear transformation to a total test score ranging from 0 to 100, with higher scores indicating better quality of life. The Swedish version of SWAL-QOL has high internal consistency, high validity, and excellent test-retest reliability. The total score will be treated and analyzed as an ordinal variable without categorization and compared between groups.
- Rate of aspiration pneumonia based on the modified criteria by Centers for Disease Control and Prevention (CDC) for stroke-associated pneumonia [25]. The data are collected through patient reports and records. The incidence of aspiration pneumonia will be compared between groups.
- Lip force [26] assessed with a device measuring the lip force in newton (N). The maximum value (best of three trials) will be treated as a continuous variable and compared between groups.
- Functional status measured with (a) the Barthel index (BI) [27] which is an ordinal scale that consists of 10 items measuring a person’s functional independence on activities in daily living (ADL): bowels, bladder, toileting, bathing, eating, dressing, grooming, transfer, stairs, and ambulation. The scale ranges from 0 to 100. BI is widely used within both stroke care and research, and it is sensitive enough to detect improvement in function after stroke as well as having good reliability between administrators. Functional status is also measured by (b) the modified Rankin Scale (mRS) [28], which is a 7-point scale ranging from 0 (no symptoms) to 6 (death). The mRS has shown high validity and reliability when tested among patients with stroke. BI and mRS total scores will be analyzed as ordinal variables without categorization and compared between groups.
- Death: The mortality rate will be compared between groups.

### Harms {17}

Although no significant adverse events are anticipated during the trial, there may be instances of muscle pain affecting the orofacial and pharyngeal muscles after training with an oral device. All adverse events, including both expected and unexpected events, will be collected throughout the study period. Harms will be collected systematically at the end of the treatment period using structured questioning, and non-systematically through spontaneous reporting if participants contact the site investigator during the trial. At study initiation, participants will be instructed to promptly notify the site investigator by telephone if any adverse event occurs.

Investigators at each study site will document the nature, severity, duration, and relatedness of all reported adverse events. Adverse events will be coded according to the Medical Dictionary for Regulatory Activities (MedDRA) and graded according to Common Terminology Criteria for Adverse Events (CTCAE) to ensure consistent classification, analysis, and reporting across sites.

In the event of an adverse occurrence, the trial investigators will report the information to the project coordinator, principal investigator, and trial steering committee. The steering committee will subsequently notify the data monitoring committee, along with a tailored action plan. All adverse events collected during the trial, including non-serious and expected events, will be summarized and reported in study publications. Events classified as related and unexpected serious adverse events (RUSAEs) will be reported in accordance with regulatory requirements.

### Participant timeline {18}

**Fig. 3:**
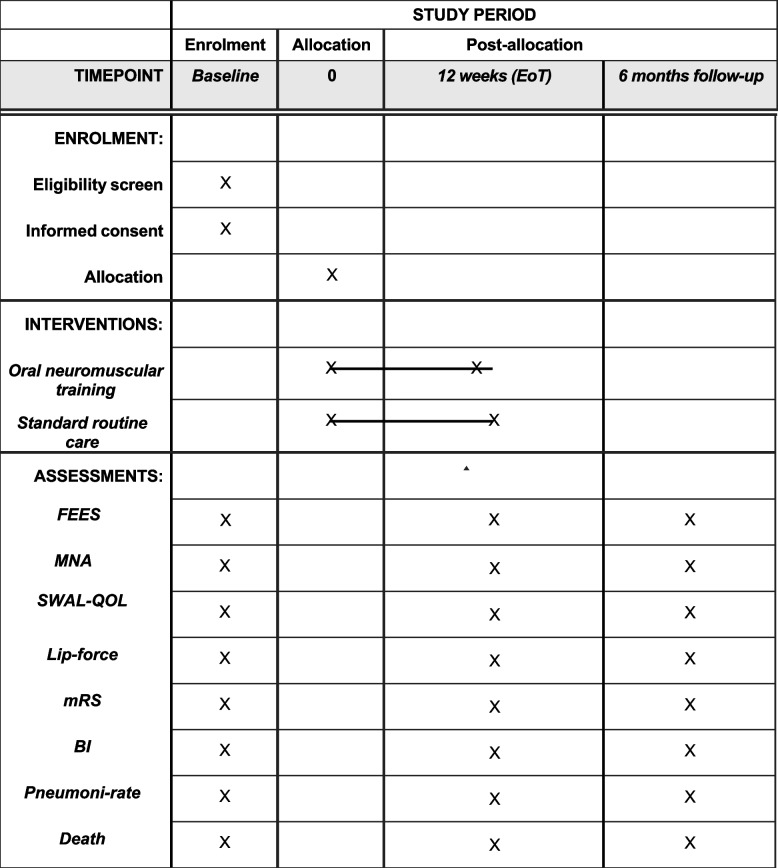
Schedule of enrollment, interventions, and assessments. Abbreviations: EoT = end of treatment; FEES = Flexible Endoscopic Evaluation of Swallowing; MNA = Mini Nutrition Assessment; SWAL-QOL = Swallowing Quality of Life Questionnaire; mRS = modified Rankin Scale; BI = Barthel index

A schematic diagram of participant enrollment, interventions, assessments, and visits for participants is presented in Fig. 3. Each participant is involved for approximately 9 months (12 weeks intervention and 6 months follow-up period) (see Fig. 3). As a standard procedure, and according to national stroke guidelines, all patients with dysphagia in the acute phase of stroke are followed up with a FEES at 1–3 months after stroke onset. If persistent dysphagia is present, the patient is invited to participate in the study. After their written informed consent is obtained, the participant will be allocated to either intervention or control group. Based on the baseline FEES assessment (performed prior allocation, as part of routine procedure), the participant will receive individualized compensatory strategies. Assessments regarding secondary outcomes will be conducted after the participants have given their informed consent. Same assessments as at baseline are performed after 12 weeks (end-of-treatment) and 6 months later (follow-up). The first participant was enrolled on 26 February 2022, and the final participant is expected to be enrolled in July 2028, based on an estimated annual enrollment rate of 84 participants for the remaining study period.

### Sample size {19}

Expected proportion of patients with dysphagia (i.e., DOSS ≤ 5) at follow-up in the control group is 65% [5]. We have dimensioned our study to detect a marginal odds ratio of 2 when using an ordinal proportional odds model. This corresponds to a proportion of patients with swallowing dysfunction of 52% in the intervention group. We expect a majority of patients to have a DOSS score of 5 or 6 at 12 weeks (end-of-treatment), based on clinical experience. Under the assumption of a marginal DOSS score distribution of 3%, 7%, 10%, 13%, 24%, 30%, and 13% for the DOSS score points of 1–7, respectively, 135 subjects are sufficient to detect an odds ratio of 2 with 90% power using an ordinal proportional odds regression model. Power calculations were performed using the function *popower*, from the R package Hmisc [29]. In the study, the odds ratio will be conditioned on the patients’ baseline DOSS score, as well as for the stratifying variables center and aspiration, which should provide further power compared to estimating a marginal odds ratio, as assumed in the power calculations. To account for a dropout rate of 20%, 168 patients will be recruited for each group.

### Recruitment {20}

Strategies for achieving adequate participant enrollment to reach target sample size are based on (a) national recruitment of several participating hospitals/sites to ensure a large number of eligible participants; (b) environment for the assessments: portable FEES equipment is accounted for to enhance inclusion of participants with the ability to perform assessments in the patient’s own home or at a healthcare center close to the patients’ own home; and (c) person devoted to the project for inclusion, assessments, randomization, and follow-ups.

## Assignment of interventions: randomization

### Sequence generation: who will generate the sequence {21a}

The random allocation sequence was generated by the study statistician (PL), who has no involvement in participant recruitment.

### Sequence generation: type of randomization {21b}

The allocation sequence was created centrally using computer-generated random numbers stratified for center and penetration-aspiration (yes/no) according to the Penetration Aspiration Scale (PAS) [30], allocating eligible patients in a 1:1 ratio to intervention or control group. Permuted blocks with random block sizes will be used.

### Allocation concealment mechanism {22}

The allocation sequence was uploaded into the REDCap (Research Electronic Data Capture) [31] randomization module. The investigators on each site will automatically receive an allocation decision upon registering a new participant into REDCap.

### Implementation {23}

The allocation sequence was generated by study statistician and contributor to the study protocol (author PL), who is not otherwise involved in the outcome assessments or intervention. A trained investigator with multifactor authentication to access the REDCap system [31] will enroll participants. The group assignment (intervention group or control group) of each participant is retrieved from REDCap after completing baseline measurements.

## Assignment of interventions: blinding

### Who will be blinded {24a}

The evaluators (speech-language pathologist and/or otolaryngologist) responsible for assessing the primary outcome (swallowing function) will remain blinded to the trial participants’ details, including the timing of the assessment and their allocation. The biostatisticians conducting the analyses will be blinded to group allocation.

### How will be blinding be achieved {24b}

At study completion, before being assessed by evaluators, FEES recordings (video clips) will have all patient-identifiable information removed, including patient name, demographics, assessment time-point, and group allocation. These pseudonymized FEES video clips will then be placed in a random order and assigned a numerical code by a staff scientist otherwise not involved in the project. Evaluators will assess the swallow function from the FEES video recording on the DOSS scale [18, 19].

### Procedure for unblinding if needed {24c}

Not applicable, as this is an open-label trial with blinded evaluators where double-blinding to intervention is not possible.

## Data collection and management

### Plans for assessment and collection of outcomes {25a}

All patients will undergo the same set of assessments at baseline, end-of-treatment, and at the 6 months follow-up. The outcome assessments will include dysphagia severity assessed with DOSS [18, 19], and PAS [30] using FEES, nutritional status [23], swallowing-related quality of life [24], rate of aspiration pneumonia, lip force [26], functional status, and health status [27, 28] including death. Data regarding stroke severity and type will be collected. All instruments that will be used have high validity and reliability (see Outcomes section). The investigators conducting the assessments will be trained in how to perform assessment with each instrument. Data collection form is in the protocol and will be added in the REDCap system [31]. The study protocol (paper format) will be stored in a secure room at Umeå University.

### Plans to promote participant retention and complete follow-up {25b}

Several strategies will be used to promote participant retention and complete follow-up. During the follow-up period, participants in both groups will be contacted 1 week after allocation for follow-up on their usual care regarding individualized compensatory strategies. The intervention group will also be followed up regarding their oral neuromuscular training (see “Intervention”) and asked if they have any questions or need assistance with monitoring the training. This keeps the participants in both groups engaged to follow the study procedures, promoting successful retention.

### Data management {26}

A data management plan (DMP) has been developed at Umeå University using DMPonline, a digital service provided by Umeå University Library, in accordance with institutional and funder requirements. The DMP outlines the procedures for data handling, storage, and analysis during the trial period and after it is finalized to ensure compliance with ethical and regulatory standards. It emphasizes the importance of data confidentiality and participant privacy, detailing how personal identifiers will be removed from the datasets before analysis.

Data collection will be carried out by using REDCap [31], as previously stated. REDCap is a secure, web-based software platform designed to support data capture for research studies. Training sessions will be conducted for all personnel involved in data collection and management to ensure that they are familiar with REDCap’s functionalities and the specific protocols of the study. This will include guidance on accurate data entry, adherence to the study timeline, and the importance of maintaining the integrity of the data throughout the research process.

### Confidentiality {33}

This trial will ensure that all participant information remains secure and accessible only to authorized personnel. Access to the data will be strictly regulated, only designated researchers are allowed to analyze the data for future studies or reports. Any publication resulting from this research will focus on aggregated findings, ensuring that no individual participant’s information is disclosed or can be inferred.

The processing of personal data will be carried out in accordance with the General Data Protection Regulation (GDPR). Participant data will be pseudonymized and stored in the REDCap system. The trial investigators will access REDCap through the Umeå University server using multifactor authentication for data collecting. Participant code numbers will be stored separately in a locked safe at the Department of Clinical Science, Unit for Speech-Language Pathology, Umeå University. FEES assessments will be stored on a secured server at Umeå University, Department of Clinical Science.

## Statistical methods

### Statistical methods for primary and secondary outcomes {27a}

The primary endpoint will be analyzed using an ordinal regression (proportional odds) model, adjusting for baseline DOSS [18, 19] as well as for the stratifying variables center and aspiration. The choice of using random or fixed effects for center is yet to be determined depending on the number of centers that will be recruited. A statistical analysis plan will be established before data retrieval, predetermining details on the analyses.

Further, nutritional status [23], swallowing-related quality of life [24], Barthel index [27], and mRS [28], all variables on ordinal scale, will be analyzed using ordinal regression, adjusted for baseline values, while group differences in the rate of aspiration pneumonia will be tested using negative binomial regression.

### Who will be included in each analysis {27b}

The primary analysis will be based on an intention-to-treat approach, including all randomized individuals. Secondary analysis will be based on a per-protocol population. Each oral device contains an integrated sensor that records training frequency, providing objective data to form the per-protocol population. Specific criteria for adherence to the intervention protocol will be specified in the statistical analysis plan. Possible confounders for the per-protocol analysis will be identified using a Directed Acyclic Graph in the statistical analysis plan (SAP), along with a definition of the per-protocol population.

### How missing data will be handled in the analysis {27c}

We will distinguish between informative and non-informative missing data for the primary outcome (DOSS).

Informative missingness: If a DOSS score is missing, a blinded investigator will assess whether the absence is caused by severe swallowing dysfunction or frailty preventing the examination. In such cases, a DOSS value of 0 (worst outcome) will be imputed, in accordance with a composite variable strategy [22]. This assessment will be based on the following criteria: a clinical swallowing examination where a Function Oral Intake Scale score of 1 is achieved [32]. To preserve the investigator’s blindness during this assessment, participants will be strictly instructed not to reveal their group allocation.

Remaining missing values will be imputed using Multivariate Imputation by Chained Equation, conditioned on patients’ baseline variables deemed to possibly be involved in a missing at random mechanism. These will be specified in the SAP.

### Methods for additional analyses (e.g., subgroup analyses) {27d}

Pre-specified subgroup analyses will be conducted to evaluate the consistency of the treatment effect across key baseline characteristics. The defined subgroups include sex, age group (<65 vs. ≥65 years), stroke type (ischemic vs. hemorrhagic; supratentorial vs. infratentorial), and baseline severity of dysphagia. To assess heterogeneity of the treatment effect, we will fit separate ordinal regression models for each subgroup factor. These models will include an interaction term between the randomized intervention and the subgroup variable. A significant interaction term will indicate a differential treatment effect across the subgroup categories.

### Interim analyses {28b}

An interim analysis is planned once 50% of participants have been assessed at end-of-treatment. At that point, an independent statistician will perform a blinded non-comparative sample size re-estimation. The updated sample size will be determined based on the observed marginal DOSS score distribution in the control group, targeting 90% power to detect a group difference at an odds ratio of 2.0, using the same sample size calculations as previously described. Because this re-estimation is non-comparative, it does not inflate the nominal type I error rate.

A second interim analysis will be conducted when 75% of the participants (according to sample size determined by the sample re-estimation) have completed the end-of-treatment assessment to evaluate efficacy. A group sequential design will be employed using the Lan-DeMets alpha-spending function with an O’Brien-Fleming type boundary. The primary ordinal regression analysis will be performed on the available data. The trial may be stopped early if the intervention shows overwhelming superiority, as determined by the boundary.

Strict operational safeguards regarding data access will be maintained throughout the study. Results of the interim analyses (both the sample size re-estimation and the efficacy calculation) will be known only to the independent statistician, while principal investigators and the study statistician will remain blinded to the results unless the independent statistician recommends stopping the trial.

### Protocol and statistical analysis plan {5}

The anonymized data will be available to other researchers from the principal investigator upon reasonable request following receipt of a written application and proposal for use of the data, approval by the Steering Committee, and establishment of a data sharing agreement.

## Oversight and monitoring

### Composition of the coordinating center and trial steering committee {3d}

Umeå University is the coordinating center for the trial and is the primary workplace of the principal investigator (first author PH), the co-principal investigator (last author PW), and the trial coordinator. The Trial Steering Committee (TSC) comprises a multidisciplinary team of academic and clinical experts. The committee includes professors, associate professors, and an assistant professor, with clinical expertise in speech-language pathology, cardiovascular medicine, neurology, otorhinolaryngology, radiology, and biostatistics, representing the three participating sites: Umeå University, Danderyd Hospital, and Södersjukhuset. The TSC are responsible for the clinical setup of the trial, ongoing management, study promotion, and planning for the interpretation and dissemination of results. Prior to the initiation of the trial, the TSC met on a daily and monthly basis, and will continue to meet monthly throughout the trial. A senior advisory group were also consulted in the planning of the trial and will be consulted on occasion throughout the trial. The trial coordinator maintains daily communication with participating sites and investigators, providing support on various aspects such as the study protocol, eligibility criteria, and REDCap monitoring. The trial coordinator and principal investigator will conduct monthly meetings with all trial investigators to ensure alignment/coherence and monitor progress.

### Composition of the data monitoring committee, its role and reporting structure {28a}

Data monitoring will be conducted annually by a Data Monitoring Committee (DMC). This committee will comprise two external researchers from the Department of Clinical Science at Umeå University and one external statistician, who are independent of both the research group and funder. The DMC will report on any adverse events and monitor study progress and safety in accordance with the Consolidated Standards of Reporting Trials (CONSORT) statement.

### Frequency and plans for auditing trial conduct {29}

Regular monitoring and evaluation will be implemented to assess data quality and adherence to the data management plan. This will involve periodic audits of the data collected—both of data entered in REDCap and by site visits. Feedback sessions with the research team will further occur to address any issues or challenges that may arise during the study.

### Protocol amendments {31}

Major changes to the protocol, including alterations to eligibility criteria, recruitment of additional sites, or patient information materials, will undergo an ethical review process. Any changes to the protocol will be communicated from the steering committee and the principal investigator to trial investigators and (if applicable) to trial participants. Additionally, the principal investigator will also communicate important protocol amendments to trial registrations such as ClinicalTrials.gov.

### Dissemination policy {8}

The progress, completion, and publication of the trial findings will be endorsed to all partners. Final data analysis will be conducted using appropriate statistical methods, with results being shared with participants, healthcare professionals, and the public in a transparent manner. The findings will be disseminated through academic publications and presentations, ensuring that the contributions of all participants and the integrity of the collected data are respected and acknowledged.

## Discussion

The DESIRE trial aims to investigate the effect of oral neuromuscular training on post-stroke dysphagia using a high-quality multicenter, national, investigator-initiated, randomized, parallel-group, superiority trial design.

The DESIRE has the potential to provide solid evidence to guide treatment decisions in the future. Dysphagia is a common and serious complication following stroke, with significant clinical and economic consequences [6, 8, 33]. Despite the significant impact of post-stroke dysphagia, there are currently no proven effective treatments available [13]. The DESIRE project aims to investigate oral neuromuscular training with an oral device as a potential solution. The oral neuromuscular training approach used in this study seeks to restore muscle and sensory function, with the goal of improving swallowing safety and efficiency.

The primary outcome measure is the degree of dysphagia, as assessed by the validated scale DOSS for FEES [19]. This scale evaluates and integrates the physiological swallowing impairment and the functional assessment of a patient’s eating and drinking in their daily living. The DOSS should thereby be considered a clinically significant endpoint rather than an intermediary one. Secondary outcomes include nutritional status, swallowing-related quality of life, rate of aspiration pneumonia, lip force, functional status, and mortality. The choice of these outcomes is based on the rationale that improved swallowing function should enable a safer and more efficient intake of food, liquids, and medications, leading to better nutritional status, reduced complications, and enhanced quality of life.

The timing of the 12-week primary and secondary endpoint is based on the rationale that, while improvements have been observed after 5 weeks of training [14, 15], the clinical experience and patient perspectives suggest that 12 weeks of training is optimal to achieve better swallowing outcomes. This is expected to result in reduced tube feeding and diet restrictions, as well as enhanced swallowing-related quality of life for participants.

The project group has good competence, equipment, personnel, infrastructure, and ethical and regional approval to conduct the study. There is also a sufficiently large base of eligible participants. The anticipated obstacles are related to (a) dropout; therefore, the estimated power calculation is based on 90% power and risk of a 20% dropout rate, and (b) compliance in conducting clinical trials on interventions; therefore, a sensor that registers the training frequency will be attached to each oral device.

If the oral neuromuscular training intervention proves effective, it could have a substantial impact on stroke care and management. Improved swallowing function could reduce the incidence of complications like aspiration pneumonia, decrease healthcare costs, and enhance patient outcomes and quality of life. The findings may also influence national and international guidelines for treatment of post-stroke dysphagia, potentially leading to improved standards of care for stroke survivors.

The high-quality trial design, comprehensive outcome measures, and thorough consideration of potential challenges make this a well-designed study with the potential for significant clinical impact for patients with dysphagia after stroke.

## Trial status

The DESIRE trial is being conducted in accordance with protocol version 4.0 (dated December 16, 2025). Enrollment commenced on July 26, 2022, with the final participant expected to be recruited by June 2028. Study completion, including final follow-up assessments, is anticipated by end of Q1 2029.

## Data Availability

No datasets were generated or analysed during the current study.

## Abbreviations

PROBE: Prospective, randomized, multicenter, open-label trial with blinded evaluators
FEES: Flexible Endoscopy Evaluation of Swallowing
PAS: Penetration Aspiration Scale
DOSS: Dysphagia Outcome Severity Scale
MNA: Mini Nutritional Assessment
SWAL-QOL: Swallowing Quality of Life Questionnaire
mRS: Modified Rankin Scale
BI: Barthel index
REDCap: Research Electronic Data Capture
GDPR: General Data Protection Regulation
TSC: Trial Steering Committee
DMC: Data Monitoring Committee
DMP: Data management plan
MedDRA: Medical Dictionary for Regulatory Activities
CTCAE: Common Terminology Criteria for Adverse Events
RUSAEs: Related and unexpected serious adverse events
